# Antibacterial Activity of *Ocimum tenuiflorum* against Drug Resistant Bacteria Isolated from Raw Beef

**DOI:** 10.4014/jmb.2409.09028

**Published:** 2025-03-26

**Authors:** Tasmina Tabassum, Anti Islam, K M Salim Andalib, Barnali Sarker, Mijan Mia, Khondoker Shahin Ahmed, Hemayet Hossain, Ahsan Habib

**Affiliations:** 1 Faculty of Agriculture, University of Bonn, Germany; 2 Institute for Integrated Studies on the Sundarbans and Coastal Ecosystems (IISSCE), Khulna University, Bangladesh; 3 Biotechnology and Genetic Engineering Discipline, Life Science School, Khulna University, Khulna, Bangladesh; 4 Pathology and Translational Pathobiology Varsity, LSU Health Shreveport, USA; 5 Bangladesh Council of Scientific and Industrial Research (BCSIR), Dhaka-1205, Bangladesh

**Keywords:** Beef samples, drug-resistant bacteria, plant extracts, antibacterial activity, HPLC analysis, molecular docking

## Abstract

Recent empirical evidence has acknowledged raw meat, particularly beef, as a significant reservoir for diverse foodborne pathogens and drug-resistant strains, posing severe threat to consumer health. This study aimed to isolate and identify drug-resistant bacteria from raw beef samples, obtained from different butcher shops in Khulna city, Bangladesh, as well as, to determine their susceptibility pattern against *Ocimum tenuiflorum* extracts. Raw beef samples were randomly collected from various butcher shops, followed by the initial isolation of thirty pure bacterial isolates. Later, 16S rRNA gene amplification and analysis identified twelve distinct bacterial species from those isolates. The antimicrobial susceptibility test results revealed ten of the isolates, including *Klebsiella pneumoniae*, *Aeromonas veronii* and *Enterobacter hormaechei*, to exhibit multidrug resistance pattern. Amoxicillin, nitrofurantoin, and flucloxacillin were found to be ineffective against most isolates. However, the ethanolic extracts of *O. tenuiflorum* were found effective in inhibiting the growth of eight species at three different concentrations. Subsequent HPLC analysis of *O. tenuiflorum* reported the presence of five secondary metabolites epicatechin, syringic acid, rutin hydrate, p-coumaric acid, and myricetin as potent contributors to the observed antimicrobial activity. Lastly, *in silico* binding interaction simulations of the secondary metabolites against five relevant targets predict syringic acid and myricetin to have effective antibacterial properties, primarily mediated by better binding affinity and molecular interactions. Thus, this study identified diverse drug-resistant bacteria in raw beef and provided novel insights into the antibacterial properties of *O. tenuiflorum* extracts.

## Introduction

Antimicrobial resistance, the next global pandemic, has been a hot topic of research in the field of microbiology, medicine and novel drug development for many decades [1]. The initial extensive evaluation of the worldwide repercussions of antimicrobial resistance reveals that the presence of resistance led to 1.27 million fatalities in 2019, surpassing the mortality rates of HIV/AIDS or malaria [2]. Moreover, projections indicate that this figure could escalate to a staggering 10 million deaths by the year 2050 [3]. The decreasing potency of antibiotics, emergence of untreatable strains, selective mutations of pathogens have pushed the humanity towards the post-antibiotic era [4]. The reasons behind being resistant are quite puzzling but the consequences exhibit an indiscriminate impact on individuals across the globe.

The emergence of multidrug resistant (MDR) bacteria is constantly jeopardizing the entire world health system, hospital management, and economic development [5]. Our globalized food chain is not an exception either, especially animal-derived foods which are the primary means of spreading antibiotic-resistant zoonotic pathogens [6, 7]. The widespread and routine use of antimicrobials in cattle feed for the purpose of promoting growth raises a significant public health concern [8]. This is particularly alarming when the same classes of antimicrobials are being used in humans as well, that can significantly boost up the prevalence of MDR microorganisms in our day to day food and meal [9].

Meat obtained from animal sources offers a rich supply of proteins, essential minerals, and vitamins, making it a valuable component of human nutrition [10]. In terms of global consumption, beef is ranked third among meats accounting for approximately 25% of total meat production [11]. The Indian sub-continent is one of the largest beef producers in the world, produced approximately 4.4 million metric tons and consumed about 2.8 million metric tons carcass weight equivalent of beef in 2022 [12]. While Bangladesh is one of the largest Muslim populated countries, beef has been a prominent feature on the menu for a long time [13]. Raw meat, while being a valuable protein source for human consumption, is also recognized as a reservoir for various foodborne pathogens [14]. A large number of studies confirmed the presence of MDR pathogens in meat products, including, *Campylobacter* species, *Salmonella* species, *Enterococcus* species, and *Escherichia coli* [14-17]. However, the prevalence of MDR pathogens in context of meat supply in Bangladesh still remains in shadow. This highlights the urgent need for comprehensive measures to address this issue and mitigate the risks associated with antimicrobial resistance. Hence, this study made an attempt to examine the prevalence and resistance properties of bacterial spectrums derived from raw beef samples, obtained from various butcher shops in Khulna, Bangladesh.

In response to this rapidly evolving and life-threatening phenomenon, the need for safe and targeted alternatives to conventional antibiotics becomes an urgent need [15, 18]. The expeditious progression of biomedical research has quenched the thirst and revealed a number of promising replacements including plant-based substances, antibodies, probiotics, bacteriophages, and antimicrobial peptides or proteins that are currently undergoing clinical trials [19]. Natural derivatives from plant sources including phytochemicals, secondary metabolites, phenols, tannins, alkaloids, steroids and flavonoids have always surprised us with their super power to cure diseases [20]. These products have been extensively valued for their medicinal properties including anti-bacterial, anti-inflammatory, anti-oxidant, anti-cancer and many more [21, 22]. Numerous studies proposed and substantiated the potential of natural products as powerful therapeutics against pathogenic bacteria. These findings provide compelling evidence that natural products can alleviate the burden of antibiotic treatments [23-25]. Consistently, researchers have spent enormous time to list out the plants and their products that have been exceptionally effective against MDR bacteria [26, 27].

From the ancient age, Tulsi (*Ocimum L*., Holy Basil) has been the center of attention of Indian medicine practitioners because of its magical therapeutic potentials [28, 29]. Tulsi has long-standing recognition for its antioxidant properties, as well as its ability to inhibit COX2 [30]. It provides protection against radiation poisoning and studies have also revealed its potential in mitigating hyperlipidemia and exerting cardioprotective effects in rat model [31]. Additionally, Tulsi has been observed to enhance immune system function, further contributing to its therapeutic potential [32]. Antimicrobial activities of Tulsi have sparked significant interest among researchers in the fields of medicinal and pharmacological sciences [33]. Recent studies have confirmed its remarkable effectiveness against methicillin-resistant *Staphylococcus aureus* [34]. Thus, this prompts the notion that Tulsi extracts may also be effective against other MDR bacterial strains as well.

Therefore, we designed this research to isolate and identify MDR bacterial strains from raw beef source, and evaluating their susceptibility to ethanolic extracts of *Ocimum tenuiflorum* (local name tulsi). Following that, we have also attempted high performance liquid chromatographic (HPLC) experiments to identify the secondary metabolites from the extracts that may exhibit antimicrobial properties. Lastly, a rigorous computational screening was employed to predict the potential antimicrobial mechanisms of these identified secondary metabolites.

## Methods

**Fig. 1:**
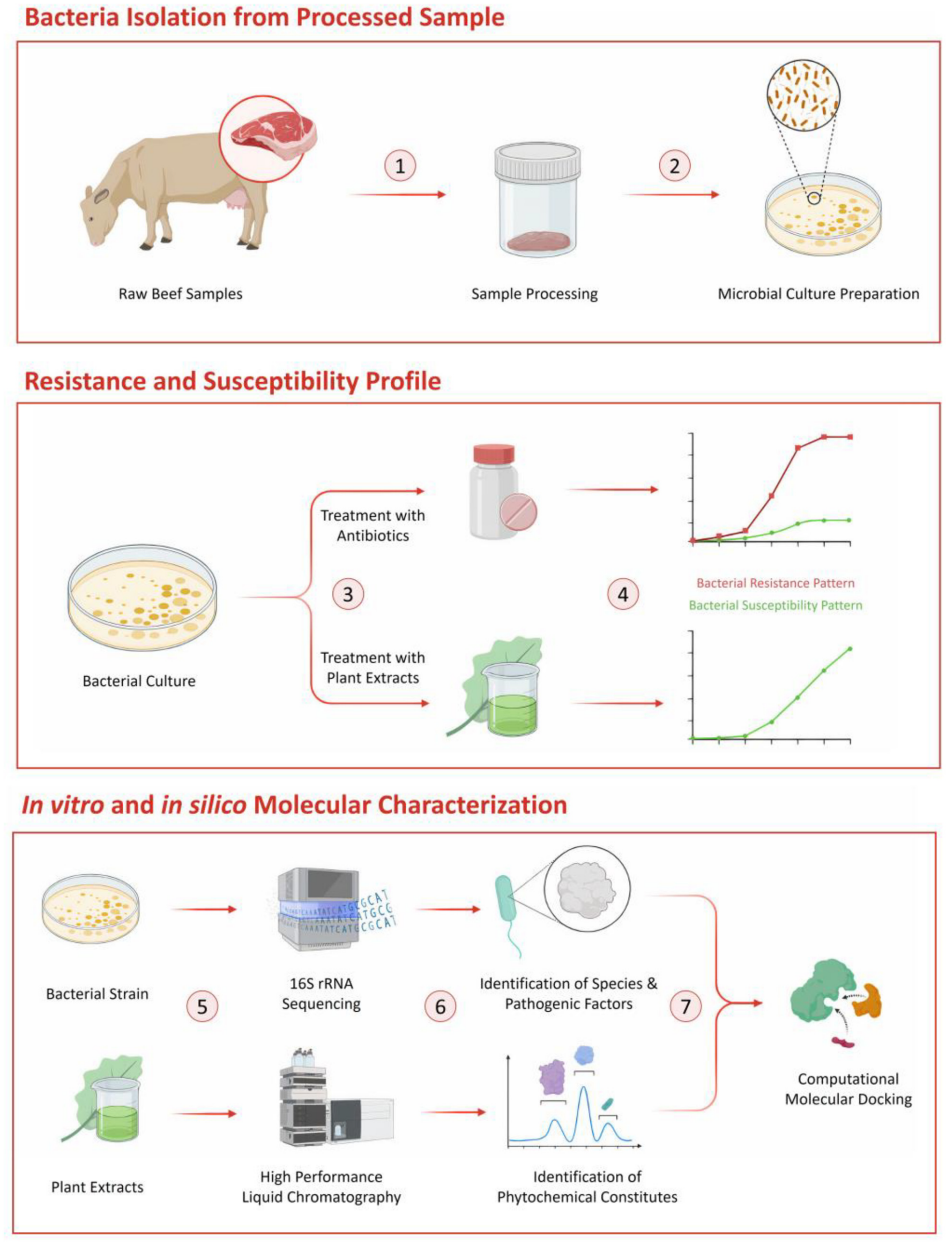
Overview of experimental workflow.

For convenience, an outline of the study methodology has been presented in Fig. 1. Brief overviews of the methods used are provided here, while additional information for replication purposes can be found in the supplementary files.

### Collection of Meat Samples

A total of thirty raw beef meat samples were collected from different butcher shops of Khulna city, Bangladesh. The selection criteria for the designated butcher shops included direct retail of meat and meat products to consumers and operating on a daily basis within well-defined geographical locations. To ensure unbiased participant selection, random tables were employed to randomly identify the individuals involved. Furthermore, the convenient sampling strategy was employed to randomly sample raw beef meat from the selected butcher shops [35]. Approximately 100 grams of dry and clean meat samples were collected using sterile zip-locked polythene bags. The samples were promptly placed in a cooling-box for transportation and stored at 4°C.

### Processing of Samples

A sterile knife was used to cut the samples aseptically into thin smaller slices, and the pieces of the five samples were mixed evenly once they had been sliced. One gram of each sample was then weighted out and homogenized (Masticator, IUL Instruments) for 3 min before being suspended in 250 ml of buffered peptone water (Condalab, Spain) to be tested. The suspension was then incubated at 37°C for an additional night.

### Bacterial Isolation

Dilutions of the incubated processed samples and suspensions were performed in 10 ml test tubes until 1:100000 dilutions were achieved. Each dilution (1 ml) was evenly distributed on appropriately labeled nutrient agar plates using a glass rod. The nutrient agar media (Nutrient Agar, HiMedia Laboratories, India) were prepared in advance and sterilized. The plates were incubated for 24 to 48 h at 37°C. The colonies with a specified form, surface texture, and edge were picked and streaked onto a new plate at the end of the incubation period.

### Disc Diffusion Assay

This study adhered to the standardized Kirby-Bauer disk diffusion susceptibility test protocol [36]. Mueller–Hinton agar (Condalab) plates were prepared and divided into four portions using a marker pen. Using a sterilized glass spreader, 100 μl of each bacterial isolatés produced suspension were freshly distributed. A total of nine commercial antibiotic discs (HiMedia Laboratories), along with a control disc containing water, were utilized in this study (Table S1).

Four antibiotic discs were gently placed on four sections of the agar plate, while a control disc was positioned at the center. The plates were promptly covered, appropriately labeled and aerobically incubated for 24 h at 37°C. After completion of incubation, the results were observed by measuring the diameter of zone of inhibition (ZOI) in millimeter (mm) and compared with National Committee for Clinical Laboratory Standards (NCCLS) guidelines [37].

### Bacterial Molecular Characterization

Pure bacterial isolates were incubated at 37°C overnight in nutrient agar media for DNA extraction. Standard protocol was followed for extracting bacterial genomic DNA [38]. Maxwell Blood DNA Kit (Promega Corporation, USA) was used according to the manufacturer’s instruction to amplify the 16S ribosomal RNA (rRNA) sequence from the bacterial genomes [39]. We employed primers as previously described by Lane, 27F (5’-AGA GTT TGA TCM TGG CTC AG-3’) and 1492R (5’- CGG TTA CCT TGT TAC GAC TT-3’) [40]. The PCR reaction was performed using a thermocycler (Agilent Technologies, USA), following the specified conditions, including an initial denaturation step at 94°C for 2 min, followed by 35 cycles of amplification at 94°C for 45 sec, 55°C for 60 sec, and 72°C for 60 sec. A final extension step was performed at 72°C for 10 min.

The final PCR products were visualized on a 1% agarose gel to confirm the success of the PCR process. The products were stained with ethidium bromide, loaded into the gel wells, and visualized using a transilluminator AlphaImager (Alpha Innotech, USA). The amplified gene fragments were purified and sequenced with the same primers from Invent Technologies Ltd., Dhaka, Bangladesh. The obtained sequences were further compared with the 16S rRNA gene sequences of the other organisms from the NCBI GenBank database using BLAST algorithm to identify the strains, followed by the submission of the sequences.

### Collection and Preparation of Plant Materials

*O. tenuiflorum* specimens were collected from the Germplasm Center at Khulna University, Khulna, Bangladesh. The specimens were carefully handled and stored in wrapped plastic sheet according to standard protocols with minor changes [41].

Fresh and healthy leaves were carefully collected, cleaned, separated from undesired materials and cut into small pieces. Plant extracts were prepared following the methods outlined by Alade and Irobi, with minor modifications [42]. After drying under sun for 3 days, 50 grams of powdered plant material were soaked in 100 ml of 50% ethanol for 8-10 days to prepare an ethanolic extract, with regular stirring every 10 h using a sterile glass rod. Once the extraction period was complete, the mixture was filtered through Whatman filter paper no. 1 (Whatman Ltd., England) to obtain a purified extract. Obtained filtrates were evaporated under reduced pressure by rotary evaporator at 50°C and air dried at room temperature. The extracts were weighted and stored at 4^0^C.

### Antimicrobial Activity of *O. tenuiflorum* Extract

The Kirby-Bauer disk diffusion susceptibility test protocol was repeated to assess the antibacterial properties of the prepared plant extract against the previously obtained bacterial isolates [43]. We prepared a stock extract at a concentration of 10 mg/ml. Small sterile filter paper discs (diameter of 6 mm) were prepared with three different volumes of 80 μl/disc, 100 μl/disc, and 120 μl/disc and gently placed on petri dishes containing nutrient agar media, which were freshly treated with 100 μl of bacterial suspension saline. Disc with phosphate-buffered saline adjusted to pH 7.4 were also included as negative control. Subsequently, the plates were incubated at 37°C for 16-24 h and antimicrobial activity was assessed by measuring the diameter of ZOI in mm.

### High-Performance Liquid Chromatography Analysis

A total of 16 phenolic compounds were taken as standard for this analysis (Table S2) and the secondary metabolites identified from the *O. tenuiflorum* extract were based on these available standards. The standards were dissolved in methanol (Merck, Germany) to prepare stock solutions with concentration ranged from 4.0 to 50 μg/ml. These stock solutions were then mixed together and serially diluted to prepare the working standard solutions.

HPLC analysis was conducted using a Shimadzu system (LC-20A, Japan) equipped with a binary solvent delivery pump, a SIL-20A HT autosampler, a CTO-20A column oven, and an SPD-M20A photodiode array detector. The separation process utilized a Luna C18 (5 μm) Phenomenex column with dimensions of 4.6 × 250 mm, operating at 33°C. The entire system was controlled by the LC Solution software.

The mobile phase composed of A (1% acetic acid in acetonitrile) and B (1% acetic acid in water) with gradient elution: 0.01-20 min (5-25% A), 20-30 min (25-40% A), 30-35 min (40-60% A), 35-40 min (60-30% A), 40–45 min (30–5% A), and 45–50 min (5% A) was used. The sample injection volume was 20 μl, and the flow-rate was set at 0.5 ml/min. The UV detector was set at 270 nm and applied for validation of method and analysis. The mobile phase was filtered through a 0.45 μm Nylon 6, 6 membrane filter (Sigma–Aldrich, USA) and degassed under vacuum.

### Molecular Docking Simulation

To further extend the understanding of the antimicrobial activities of the *O. tenuiflorum* extract, this study incorporated a rigorous docking simulation. The pathogenic proteins from the bacteria that were highly susceptible to the plant extract were selected based on structure availability, gene essentiality and pathogenicity. The crystal structures of the proteins were either retrieved from RCSB PDB or prepared through homology modeling [44, 45]. Compounds identified from the *O. tenuiflorum* extract through chromatographic experiments were undertaken as ligand molecules and their three dimensional structures were obtained from PubChem Database [46]. Preprocessing and optimization of proteins and ligands were performed in Biovia Discovery Studio Modeling Environment v4.5, maintaining standard protocols [47].

The ligands and protein receptors were subjected to structure-based flexible molecular docking using the DockThor v2.0 Portal [48]. A receptor box measuring 40 Å × 40 Å × 40 Å was defined and the Lamarckian genetic algorithm was employed with 100,000 evaluations per run, a population size of 750, and 25 runs per ligand. The docking results were obtained from the server and subsequently analyzed and visualized in Biovia Discovery Studio Modeling Environment v4.5.

### Statistical Analysis

All experimental procedures were conducted in triplicate, and the results are presented as the mean of three replicates ± standard deviation (SD). A *p*-value threshold of < 0.05 was considered significant for the study. All statistical analyses were carried out using GraphPad Prism software (version 7; GraphPad Software Inc., USA).

## Results

### Isolation and MDR Profile of Bacterial Isolates from Beef Samples

A total of 30 pure bacterial isolates were obtained from the raw meat products sampled from different butcher shops. The isolates were classified into twelve distinct groups based on their morphological and biochemical characteristics (Table S3). Most of the isolates were rod shaped and convex in elevation. Only two flat elevated isolates were screened to be gram positive.

**Fig. 2:**
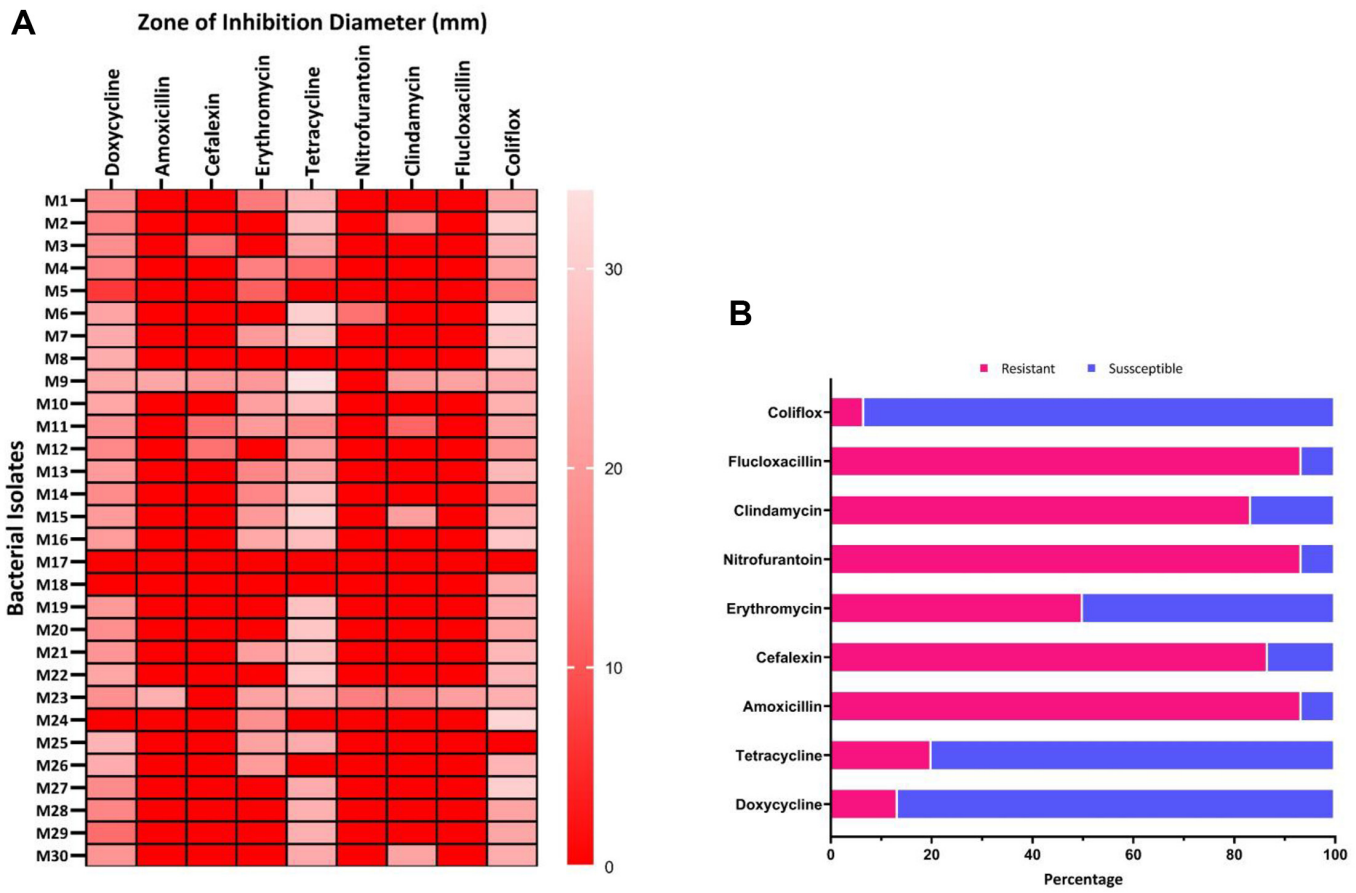
MDR profile of the 30 bacterial isolates. (**A**) A heatmap visual representation the ZOI (in mm) produced by each bacterial isolate. The values presented are the average of three replicates. (**B**) A stacked bar plot illustrating the percentage of the 30 bacterial isolates tested for nine different antibiotics, categorized as either resistant (shown in cerise) or susceptible (shown in blue).

Out of the thirty isolates examined, M17 was resistant to all the nine tested antibiotics (Fig. 2). Except for M9 and M23, all the isolates exhibited multidrug resistance pattern. While certain bacteria displayed intermediate resistance, for the purpose of classification, they were considered susceptible (Table S4). A significant proportion of the isolates were resistant to amoxicillin, cefalexin, nitrofurantoin, and clindamycin.

### Molecular Identification of the Bacteria Isolates

**Fig. 3:**
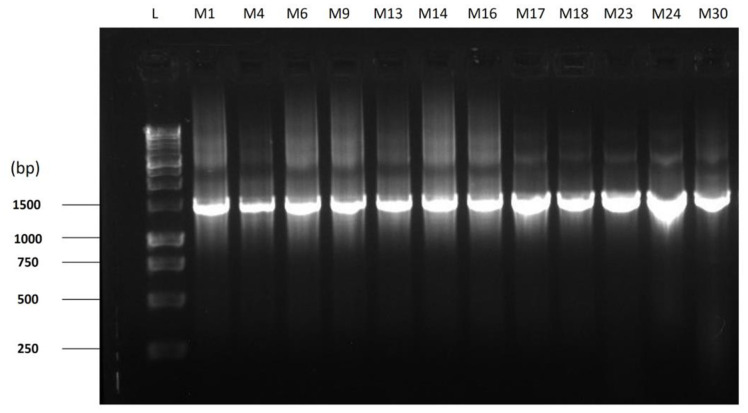
Gel electrophoresis bands. Agarose gel electrophoresis visualization of the amplified 16S rRNA PCR products from the twelve distinct bacterial groups. Lane L represents the 1 kb DNA ladder used as a size marker. The bands of high intensity correspond to the PCR amplified 16S rRNA gene of the respective bacterial isolates in the other lanes.

Genomic DNA were extracted from the twelve distinct bacterial groups and the presence of amplified 16S rRNA PCR products were detected on agarose gel electrophoresis (Fig. 3). These amplification events generated DNA amplicons of approximately 1500 base pairs in length. The alignment of these partial 16S rRNA sequences from the isolates against the NCBI GenBank database identified twelve distinct bacterial species, as shown in Table 1, with their corresponding accession numbers for the 16S rRNA sequences. Out of the twelve isolates, *Bacillus cereus* was the only one identified as gram positive, while the rest were gram negative. Four isolates were classified as *Aeromonas* species (*A. hydrophila*, *A. dhakensis*, *A. veronii*, *A. caviae*), in addition to *Klebsiella pneumoniae*, *Comamonas aquatica*, and *Enterobacter hormaechei*.

### Antibacterial Potential of *O. tenuiflorum* Extract

**Fig. 4:**
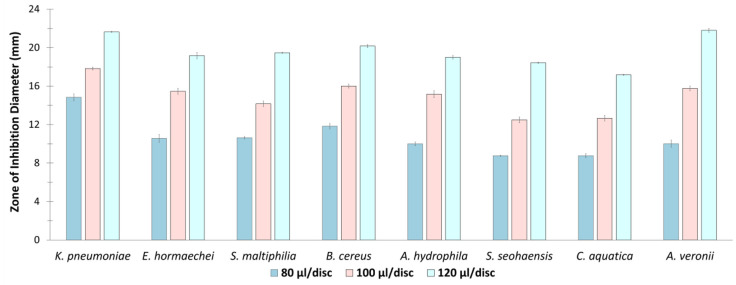
Antibacterial Potential of *O. tenuiflorum* Extract. The ZOI of ethanolic *O. tenuiflorum* extracts was measured against eight multidrug-resistant bacterial isolates. All values are significantly different at *p*<0.05

Ethanolic extract of *O. tenuiflorum* were significantly active against eight of the identified bacterial strains (Fig. 4). The ZOI diameter varied between 8.76 ± 0.05 to 14.83 ± 0.35 mm, 12.5 ± 0.3 to 17.83 ± 0.15 mm, and 17.2± 0.05 to 21.6 ± 0.05 mm at doses of 80 μl/disc, 100 μl/disc, and 120 μl/disc, respectively, exhibiting strong antibacterial property [49]. At volumes of 80 μl/disc and 100 μl/disc, *O. tenuiflorum* exhibited the strongest activity against *K. pneumoniae*, while at a volume of 120 μl/disc, the strongest activity was observed against *A. veronii*. The lowest diameter of ZOI was observed against *Shewanella seohaensis* at both 80 μl/disc and 100 μl/disc volumes, as well as against *C. aquatica* at the 120 μl/disc volume. However, the plant extract did not result in any ZOI against four bacterial strains, namely *C. jiangduensis*, *A. dhakensis*, *Metalysinibacillus saudimassilinesis*, *A. caviae*. The negative control used in this study did not yield any ZOI against any of the isolates.

### Phytochemical Contents of Plant Extract

**Fig. 5:**
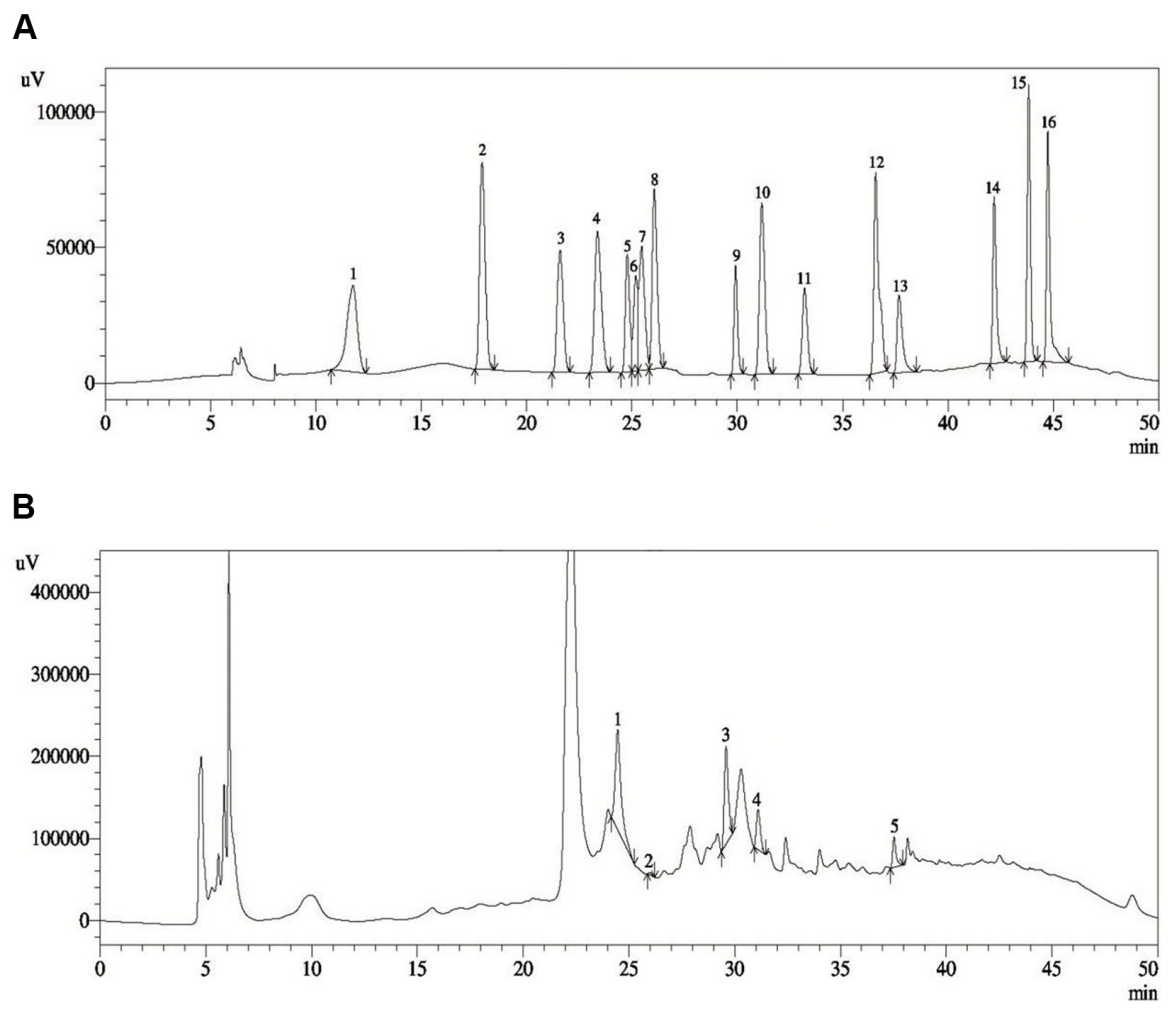
HPLC chromatogram. The HPLC fingerprint analysis revealed the presence of five peaks corresponding to five secondary metabolites in the *O. tenuiflorum* extracts. (**A**) Chromatogram of the sixteen standard compounds. (**B**) Chromatogram of the five identified compounds. The peaks were identified as follows: (1) epicatechin, (2) syringic acid, (3) rutin hydrate, (4) p-coumaric acid, and (5) myricetin.

The chromatograms from the HPLC analysis performed on the ethanolic extract of *O. tenuiflorum* are presented in Fig. 5. Among the sixteen standard compounds examined, five secondary metabolites were identified at different concentration from the extract (Table 2). The analysis revealed that Epicatechin exhibited the highest quantity among the active compounds detected, with a concentration of 308.33 ± 0.14 mg/100 g dry extract. On the other hand, syringic acid was observed to be present in the lowest concentration, with a value of 2.90 ± 0.47 mg/100 g dry extract. Additionally, Rutin hydrate, p-Coumaric acid, and Myricetin were also identified from the ethanolic extract of *O. tenuiflorum*.

### Molecular Interaction Study

**Fig. 6:**
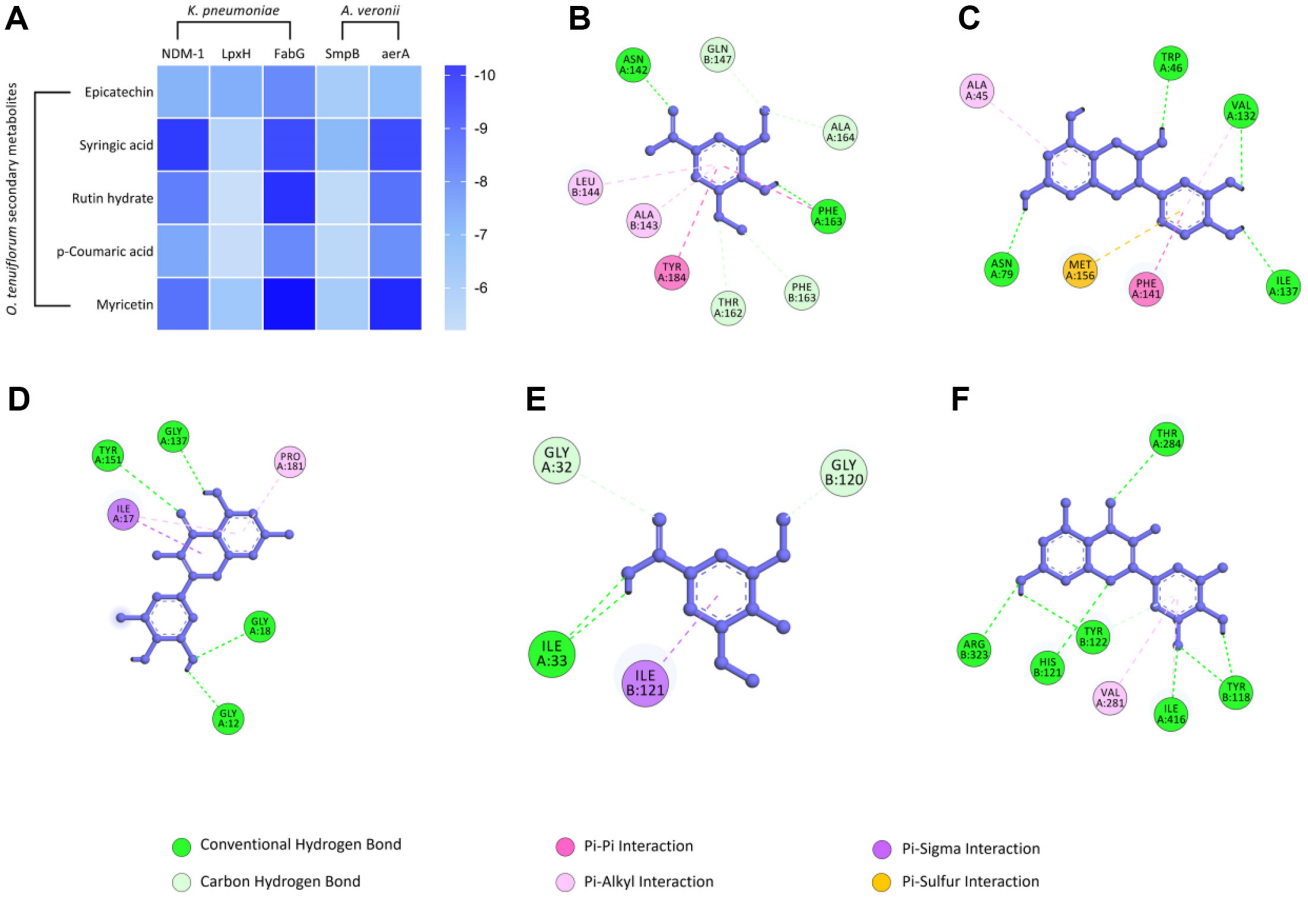
Summary results from molecular docking study. (**A**) Binding affinity values for each receptor-ligand complex has been summarized in a heatmap plot. The values are represented as kCal/mol and intensity of blue represents binding affinity, with higher intensity correlating to higher binding affinity. 2D binding orientation of the best docked compound has been visualized for (**B**) NDM-1 with syringic acid, (**C**) LpxH with epicatechin, (**D**) FabG with myricetin, (**E**) SmpB with syringic acid, and (**F**) aerA with myricetin.

In our study, we found that the ethanolic extract exhibited significant activity against eight identified bacterial strains. Notably, the strongest effects were observed against *K. pneumoniae* and *A. veronii*. Hence, this study selected three crucial proteins (NDM-1, LpxH and FabG) of *K. pneumoniae* [50] and two pathogenic factors (SmpB and aerA) of *A. veronii* [51, 52] for comprehending their molecular interactions with the five secondary metabolites of *O. tenuiflorum*. Crystal structures of NDM-1 (PDB ID: 4EXS), LpxH (PDB ID: 6PIB) and FabG (PDB ID: 6T77) were downloaded from RCSB PDB and optimized accordingly. Crystal structures of SmpB and aerA were determined through homology modeling and validated as described previously [53, 54]. The binding affinity scores of the molecular docking simulations are presented in Fig. 6, the intensity of blue represents binding affinity, with higher intensity correlating to higher binding affinity. Epicatechin demonstrated moderate interaction with all three proteins of *K. pneumoniae*, while rutin hydrate exhibited the lowest binding affinity with LpxH (-5.2 kCal/mol). Notably, myricetin demonstrated the strongest interaction mediated by four conventional hydrogen bonds and obtained the highest affinity score (-10.2 kCal/mol) when docked against FabG.

All the secondary metabolites exhibited strong interactions with aerA proteins of *A. veronii*. Particularly, syringic acid exhibited the most promising overall inhibitory actions against both proteins. Again, myricetin displayed the highest interaction strength (-9.7 kCal/mol) driven by seven conventional hydrogen bonds with the aerA protein. On the other hand, rutin hydrate exhibited the lowest binding affinity against SmpB protein (-5.5 kCal/mol).

## Discussion

The indiscriminate use of antibiotics in livestock feed, the subsequent microbial contamination of raw beef across the distribution chain and long storage at ambient temperature can significantly contribute to the emergence and dissemination of resistant bacteria [55]. This molecular investigation focused on detection and identification of twelve resistant bacteria, isolated from raw beef samples obtained from different butcher shops. Likewise, an earlier study has also identified the presence of pathogenic resistant bacteria in poultry farms and chicken meat samples in Bangladesh [56, 57]. These findings highlight a concerning issue, particularly in developing countries like Bangladesh, where butcher or meat shops often face challenges in maintaining adequate hygienic conditions during meat processing.

In the present study, four *Aeromonas* spp. emerged as the predominant isolates. These estuarine pathogenic bacteria are commonly found in aquatic environment, fish, meats, and fresh vegetables [58]. They have been implicated in a wide range of human diseases, including gastroenteritis, chronic diarrhea, wound infections, urinary tract infections, and septicemia [59]. *Aeromonas* spp. has been identified in various studies conducted worldwide, highlighting its presence in different raw meat samples. For instance, it was identified from 47% of raw meat sample in Saudi Arabia [60], 79% of ground beef samples in eastern Canada [61], 78.5% of naturally contaminated Spanish fermented sausages [62] and 32.8% animal meat products in Egypt [63]. Among the four *Aeromonas* spp., *A. veronii* exhibited resistance to 8 out of the 9 tested antibiotics but susceptible to coliflox, making it the most prevalent resistant species. However, it is noteworthy that several studies have consistently reported *A. hydrophila* as the most resistant species, characterized by the presence of notable virulence factors [64-66].

In our study, *C. aquatica* exhibited resistance against all the tested antibiotics. This finding is consistent with previous studies that have reported the presence of multiple carbapenemase and lactamase genes, as well as multidrug-resistance plasmids, in *C. aquatica* isolates [67, 68]. This opportunistic pathogen is abundant in aquatic and soil environments and have been increasingly reported to be associated with invasive human infections [69]. The presence of *C. aquatica* in the tested meat samples suggests that contamination may have occurred due to improper handling practices, such as inadequate hygiene measures during processing and the use of unhygienic water. This study also reports the presence of *C. jiangduensis* which was resistant against four tested antibiotics. This bacterium has been previously identified in agricultural soil environments [70].

Improper storage temperatures during the transportation of meat can create favorable conditions for *B. cereus* growth [71]. It is commonly associated with emetic types of food borne diseases, as well as, diarrheal, gastrointestinal and nongastrointestinal infections [72, 73]. In this study, *B. cereus* was found to be resistant only to nitrofurantoin. In contrast, *K. pneumoniae* is not typically recognized as a foodborne pathogen. This opportunistic bacterium is prevalent in the environment and often present in the gastrointestinal tracts of animals consumed by humans [74, 75]. As a result, it can contaminate meat and lead to various extraintestinal infections, such as meningitis, bacteremia, septicemia, liver abscess, and wound infections [76]. *K. pneumoniae* isolated from the raw beef samples in this study exhibited resistance to five of the tested antibiotics. *E. hormaechei* is a zoonotic opportunistic pathogen that is naturally present in the intestinal microbiota of humans and animals and frequently found in soil and sewage water [77]. It has been detected in a wide range of food items, including animal-derived products and dried meat [78]. This isolate also demonstrated a similar gradual trend of drug resistance as *K. pneumoniae*.

Among the other isolated bacteria, *Stenotrophomonas maltophilia* is increasingly acknowledged as an opportunistic pathogen linked to nosocomial infections, chronic pulmonary infections, and various cancer-related conditions [79]. This environmental bacterium is commonly found in aquatic environments and has been previously isolated from various sources including water treatment and distribution systems, wastewater plants, washed salads, hand-washing soap, bottled/tap water, as well as leafy green vegetables [80, 81]. Both *S. maltophilia* and *S. seohaensis* demonstrated resistance to five tested antibiotics, whereas *M. saudimassiliensis* exhibited resistance solely against cefalexin. Given that the majority of the isolates are from an aquatic habitat, it can be hypothesized that the tested raw beef samples were primarily contaminated with unhygienic water. Additionally, improper storage conditions may have contributed to further contamination of the samples. Therefore, it is crucial to observe hygienic principles during livestock slaughter and subsequent meat processing.

The present study provides compelling evidence of a higher frequency of antibiotic resistance and multidrug resistance among the bacterial isolates identified from raw beef. We found that that over 90% of the isolates exhibited individual resistance to amoxicillin, nitrofurantoin, and flucloxacillin. Conversely, more than 90% of the isolates were susceptible to coliflox and over 80% were susceptible to tetracycline antibiotics. Notably, erythromycin showed a unique pattern of inhibiting 50% of the bacterial isolates while being susceptible to the other 50%. This makes it the only antibiotic with such efficacy in this study. These findings are more or less similar to other studies [82-84].

The presence of potent phytochemicals in higher plants has long been recognized for their antibacterial activity [85]. Numerous studies have reported the utilization of ethanolic plant extracts for their antimicrobial properties [86, 87]. In the present work, we assessed the antimicrobial potential of the ethanolic extract of *O. tenuiflorum* against the resistant bacterial isolates obtained from raw beef samples. The ethanolic extracts demonstrated promising antibacterial activity in terms of ZOI against majority (~ 66.7%) of isolates. Furthermore, the results indicated that the antibacterial effectiveness of the extracts increased proportionally with the concentration, demonstrating a dose-dependent effect across the tested bacteria. The antibacterial activity of *O. tenuiflorum* essential oils has been previously investigated and found to be effective against a range of bacterial species, including Proteus vulgaris, *S. aureus*, *Pseudomonas aeruginosa*, and *E. coli* [88]. The essential oils from *O. sanctum*, another subspecies of the Tulsi, have also demonstrated bacteriostatic activity at a concentration of 2.25–2.5 μg/ml against MDR *S. aureus*, *P. aeruginosa*, and *E. coli* [89, 90]. And to our knowledge, this present study represents the first report to shed light on the antibacterial properties of *O. tenuiflorum* ethanolic extract against MDR bacteria.

While the specific compounds responsible for the observed antibacterial activity have not been investigated yet, the present study identifies and reports five secondary metabolites in the *O. tenuiflorum* ethanolic extract. These secondary metabolites exhibit antibacterial effects by disrupting bacterial cell membranes, inhibiting biofilm formation, and inducing oxidative stress, leading to cell death. Chromatographic analysis of the extract exposed the prominent presence of epicatechin and rutin hydrate. Epicatechin has been shown to exhibit notable antimicrobial activities, whether in its natural extract form or when tested individually [91, 92]. Its effectiveness against various microbial pathogens highlights its potential as a natural antimicrobial agent [93]. Furthermore, epicatechin has been found to possess a protective role in cell cultures, indicating its ability to safeguard cells from any adverse effect [94]. Rutin hydrate has proven to be effective against a diverse array of bacteria, encompassing both gram-negative and gram-positive strains [95]. Notably, its antimicrobial actions have been observed against severe pathogens such as *S. aureus*, *Acinetobacter baumannii*, *P. aeruginosa*, and *Candida krusei* [96]. This demonstrates the broad-spectrum activity of rutin hydrate, making it a promising compound for combating various bacterial infections [97]. Myricetin has been found to possess robust antibacterial effects against several clinically significant organisms, including MDR *S. aureus*, MDR *Burkholderia cepacia*, vancomycin-resistant enterococci, *K. pneumoniae*, and *S. epidermidis* [98, 99]. Another component from extract, p-coumaric acid has been demonstrated to possess antibacterial properties by targeting bacterial cell membranes and binding to bacterial genomic DNA, interfering with vital cellular processes and inhibiting bacterial growth [100]. Syringic acid, the least concentrated component of the extract, has also been reported to exhibit antibacterial ability against MDR *S. aureus* and *Cronobacter sakazakii* by impacting bacterial cell membrane permeability [101]. Notably, synthetic analogs of syringic acid have also exhibited antibacterial activity against *Salmonella typhi* and *Oenococcus oeni* [102]. These findings underscore the significance that these secondary metabolites could potentially contribute to the observed antimicrobial effects *O. tenuiflorum* *ethanolic* extract.

This notion was further strengthened by *in silico* molecular docking of these secondary metabolites against specific targets or pathogenic factors associated with *K. pneumoniae* and *A. veronii*. Additionally, among the eight strains analyzed, the selected five factors NDM-1, LpxH, FabG, SmpB, and aerA are present in *K. pneumoniae*, *A. veronii*, *E. hormaechei*, and A hydrophila. Conversely, while FabG and SmpB are likely present in *S. maltophilia*, *S. seohaensis*, *C. aquatica*, and *B. cereus*, NDM-1 and LpxH are generally not associated with these organisms. The computational analysis revealed strong binding affinities among these interactions, primarily driven by conventional hydrogen bonds and carbon hydrogen bonds. The formation of these bonds between the metabolites and the target molecules contributes to the stability of the complex and may disrupt the normal functioning of the bacterial targets, potentially leading to the inhibition of pathogenic factors or vital cellular functions in the bacteria. Therefore, these *in silico* binding interactions provide valuable insights into the underlying mechanisms of the observed antimicrobial effects exhibited by the identified secondary metabolites. This knowledge can guide future pre-clinical and clinical studies to validate these interactions and further large scale *in vivo* analysis to explore the therapeutic potential of these metabolites in response drug-resistant bacteria.z

## Conclusion

The current study focused on isolating, screening, and identification of drug resistant bacteria from raw beef samples and evaluate the *in vitro* antimicrobial activity *O. tenuiflorum* ethanolic extracts, as well as, identify candidate antibacterial agents. Molecular analysis of the thirty samples identified twelve different pathogenic and opportunistic bacterial strains, dominated by *Aeromonas* spp. The susceptibility testing of these strains using nine commercial antibiotics in a disc diffusion assay revealed that ten of the isolates exhibited a multidrug resistance pattern. All the isolates were found to be resistant to at least one of the tested antibiotics, while *C. aquatica* was resistant against all the tested antibiotics. Ethanolic extracts of *O. tenuiflorum* exerted significant inhibitory properties against eight bacterial isolates during *in vitro* antimicrobial assay. Subsequent, HPLC analysis of the *O. tenuiflorum* ethanolic extracts revealed the presence five secondary metabolites as potential contributors to the observed antimicrobial effects. Following that, computational docking analyses added an extra dimensional insight into the potential interactions between the identified plant metabolites and the target molecules of the bacteria. The findings of this study highlight a concerning issue of high bacterial contamination in the tested meat samples. This raises significant concerns regarding the safety of the meat and the potential for foodborne illnesses. This study also provides novel insights into the potential antimicrobial agents present in *O. tenuiflorum* ethanolic extracts and their underlying molecular mechanisms. Finally, considering the global health concerns and the urgent need for novel antibiotic alternatives, exploring the inter-molecular complexations, interactions, and synergistic relationships between the phytochemicals present in *O. tenuiflorum* extracts is a promising avenue for future research.

## Supplemental Materials

Supplementary data for this paper are available on-line only at http://jmb.or.kr.



## Figures and Tables

**Table 1:** Identification of bacterial isolates from raw beef samples.

SL.	Isolate No.	Organism	Accession No
1	M1	*Klebsiella pneumoniae*	OR237215
2	M4	*Stenotrophomonas maltophilia*	OR229870
3	M6	*Comamonas jiangduensis*	OR229873
4	M9	*Bacillus cereus*	OR229900
5	M13	*Aeromonas hydrophila*	OR229876
6	M14	*Aeromonas dhakensis*	OR229875
7	M16	*Shewanella seohaensis*	OR229877
8	M17	*Comamonas aquatica*	OR229878
9	M18	*Aeromonas veronii*	OR229884
10	M23	*Metalysinibacillus saudimassiliensis*	OR229880
11	M24	*Aeromonas caviae*	OR229883
12	M30	*Enterobacter hormaechei*	OR229886

**Table 2:** Secondary metabolites present in the ethanolic extract of *O. tenuiflorum*.

Compound name	Retention time (min)	Concentration (mg/100 g dry extract)
Epicatechin	24.423	308.33 ± 0.14
Syringic acid	25.987	2.90 ± 0.47
Rutin hydrate	29.482	125.37 ± 1.08
p-Coumaric acid	31.081	57.41 ± 0.48
Myricetin	37.532	61.69 ± 0.02
